# Glyco-Engineering Plants to Produce Helminth Glycoproteins as Prospective Biopharmaceuticals: Recent Advances, Challenges and Future Prospects

**DOI:** 10.3389/fpls.2022.882835

**Published:** 2022-04-29

**Authors:** Alex van der Kaaij, Kim van Noort, Pieter Nibbering, Ruud H. P. Wilbers, Arjen Schots

**Affiliations:** Laboratory of Nematology, Plant Sciences Group, Wageningen University and Research, Wageningen, Netherlands

**Keywords:** glycan, glycosylation, glycoprotein, biopharmaceutical, helminth, plant made pharmaceutical

## Abstract

Glycoproteins are the dominant category among approved biopharmaceuticals, indicating their importance as therapeutic proteins. Glycoproteins are decorated with carbohydrate structures (or glycans) in a process called glycosylation. Glycosylation is a post-translational modification that is present in all kingdoms of life, albeit with differences in core modifications, terminal glycan structures, and incorporation of different sugar residues. Glycans play pivotal roles in many biological processes and can impact the efficacy of therapeutic glycoproteins. The majority of biopharmaceuticals are based on human glycoproteins, but non-human glycoproteins, originating from for instance parasitic worms (helminths), form an untapped pool of potential therapeutics for immune-related diseases and vaccine candidates. The production of sufficient quantities of correctly glycosylated putative therapeutic helminth proteins is often challenging and requires extensive engineering of the glycosylation pathway. Therefore, a flexible glycoprotein production system is required that allows straightforward introduction of heterologous glycosylation machinery composed of glycosyltransferases and glycosidases to obtain desired glycan structures. The glycome of plants creates an ideal starting point for *N*- and *O*-glyco-engineering of helminth glycans. Plants are also tolerant toward the introduction of heterologous glycosylation enzymes as well as the obtained glycans. Thus, a potent production platform emerges that enables the production of recombinant helminth proteins with unusual glycans. In this review, we discuss recent advances in plant glyco-engineering of potentially therapeutic helminth glycoproteins, challenges and their future prospects.

## Pharmaceutical Protein Production in Plants

In plant molecular farming, valuable small molecules and proteins are produced in plants often with the aim to ultimately use these compounds as drugs. Plant molecular farming emerged around 1990 after the first expression of antibodies and albumin in plants (Hiatt et al., 1989; Sijmons et al., 1990; Stieger et al., 1991). Afterward, proof-of-concept studies have confirmed the potential for plant-produced biopharmaceuticals as a substitute of or supplement to conventional drugs (Schillberg and Finnern, 2021). Plant-produced biopharmaceutical proteins include a wide variety of proteins such as antibodies, vaccines, cytokines and enzymes. Currently, two plant-produced pharmaceuticals have received market approval. Carrot-cell-suspension-produced glucocerebrosidase has been approved to be used in enzyme replacement therapy for the lysosomal storage disorder Gaucher disease (Zimran et al., 2011; Grabowski et al., 2014) and recently a plant-based SARS-CoV2 vaccine has been approved in Canada (Health Canada Medicago Covifenz COVID-19 Vaccine, 2022). Many other plant-based biopharmaceuticals are currently in clinical trials, including vaccines for influenza (Ward et al., 2020) and *Bacillus anthracis* (Paolino et al., 2022). As the field of plant molecular farming continues to develop and reaches maturity, an increasing number of biopharmaceuticals will enter clinical trials and possibly reach market approval.

**FIGURE 1:**
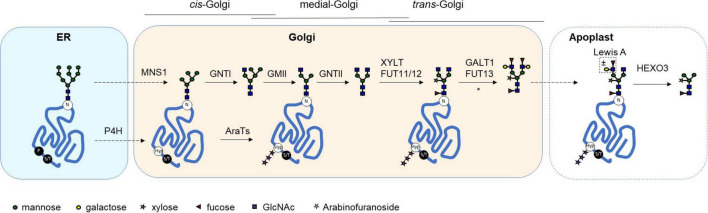
Glycosylation pathways in plants. A schematic overview of successive glycan modifying steps in the plant secretory pathway. Glycoproteins receive an *N*-glycan in the endoplasmic reticulum (ER) on an asparagine residue (N) within the consensus sequence (*N*-X-S/T), where X can be any amino acid except proline. After ER quality control, the correctly folded protein passes through the Golgi compartment. Within the Golgi immature high mannose-type *N*-glycans are modified in a tightly regulated process. First, Golgi α-mannosidase I (MNS1) trims down the *N*-glycan to a Man5 structure. In subsequent steps a diantennary *N*-glycan is synthesized, which in plants is substituted with core α1,3-fucose and β1,2-xylose. In some cases [as indicated by an asterisk (*)], this *N*-glycan is further extended with Lewis A motifs. Once secreted into the extracellular space (or apoplast) a terminal GlcNAc glycan structure can be trimmed down to a paucimannosidic glycan by the β-hexosaminidase HEXO3. Classical *O*-glycan synthesis in plants is initiated by the hydroxylation of proline residues by prolyl-4-hydroxylases (P4H) in the ER and/or Golgi. These hydroxyproline residues (Hyp) can then be substituted with extensin-like *O*-glycans (substitution with arabinofuranoside by AraTs) or more complex arabinogalactan *O*-glycans, recently reviewed in Petersen et al. (2021). In contrast to animals, serine (S) and threonine (T) residues are not substituted with *O*-glycans in plants. GnTI, *N*-acetyl-glucosaminyltransferase I; GMII, Golgi-α-mannosidase II; GnTII, *N*-acetyl-glucosaminyltransferase II; XYLT, β1,2-xylosyltransferase; FUT11/12, Core α1,3-fucosyltransferase; GALT1, β1,3-galactosyltransferase; FUT13, α1,4-fucosyltransferase.

The majority of biopharmaceutical proteins are glycosylated (Walsh, 2018), a process where carbohydrate structures (glycans) are covalently attached to a protein backbone. Glycans can be crucial for the function of these proteins and a plethora of biological processes. On protein level, glycans can be important for protein folding and glycan dependent quality control, localization, protection against degradation, solubility and activity (Moremen et al., 2012; Hebert et al., 2014; Strasser et al., 2021). In addition, glycans can play a role in protein-protein interactions, interactions with cells and tissues, and immunogenicity (Varki and Gagneux, 2015). For this reason, heterologous production of complex glycoproteins is often performed in eukaryotic expression systems that naturally glycosylate proteins. Two main types of glycosylation exist: *N*-glycosylation and *O*-glycosylation. Initial *N*-glycosylation steps occur in the endoplasmic reticulum and are highly conserved among eukaryotes (Strasser, 2018), whereas glycans are modified with kingdom-specific glycan structures in the Golgi (Strasser, 2016). In contrast, *O*-glycosylation is not that highly conserved between eukaryotes. Various types of *O*-glycosylation exist in different species, and are predominantly initiated in either the ER or Golgi depending on the type of *O*-glycan. Common steps in the glycosylation machinery of plants that affect the production of heterologous glycoproteins are illustrated in Figure 1.

Notwithstanding their different glycosylation pattern, plants have shown to be well suited for the production of human glycoproteins over the last two decades (Bosch et al., 2013; Schoberer and Strasser, 2018). More recently, glycoproteins from parasitic worms have been reconstituted in plants (Wilbers et al., 2017). The glycome of plants is limited (with the exception of *O*-glycans on cell wall glycoproteins) and this allows production of heterologous proteins with a more homogenous glycan composition. It is also striking that plants tolerate the presence of engineered exogenous *N*- and *O*-glycans on their proteins by not showing an aberrant phenotype. These two properties make plants a suitable platform for the heterologous production of glycoproteins through adaptation of their glycosylation machinery, referred to as glyco-engineering (Monter*o*-Morales and Steinkellner, 2018). Plant-production of glycoproteins is scalable from cell suspension cultures in flasks to GMP bioreactors, or entire plants in greenhouses (Lobato Gómez et al., 2021). Moreover, protein production in plants is rapid and flexible when using transient expression with agrobacterial or viral vectors (Kapila et al., 1997; Lobato Gómez et al., 2021). Plant expression systems also guarantee the absence of mammalian pathogens and oncogenic sequences (Fischer and Emans, 2000). These advantages and recent admission of the plant-based SARS-CoV2 vaccine, show that plant-based production systems are now a scalable, economically viable and safe option to produce biopharmaceuticals that depend on a specific glycan composition.

For many years the plant glyco-engineering field has focused mainly on the production of biopharmaceutical glycoproteins with a humanized glycan composition. Non-human glycoproteins, with native glycosylation patterns, have been largely ignored but form an untapped pool of potential biopharmaceuticals. The prime example of non-human glycoproteins with putative therapeutic applications are those produced by helminths (parasitic worms). Helminth glycoproteins show potential as therapeutics against immune-related disease (including allergies and autoimmune diseases) and are highly promising anti-helminth vaccine candidates (Bunte et al., 2022). Anti-helminth vaccines are direly needed since a quarter of the world population is infected with parasitic worms (Hotez et al., 2008; WHO, 2022). In addition, helminth infections in livestock cause a lot of distress by affecting animal well-being and lead to a loss in livestock productivity (Charlier et al., 2016). Once infected, anthelmintic drugs can be administered to reduce (chronic) infections of helminths in both humans and livestock. However, excessive usage, notably in animal husbandry, has led to anthelmintic drug resistance (James et al., 2009). Therefore, vaccines as alternative control measure are required to protect humans and livestock (Hotez et al., 2016; Claerebout and Geldhof, 2020). Currently, the only source to obtain helminth antigens is from the parasite itself, which requires the sacrifice of deliberately infected animals. Previous attempts to produce recombinant helminth vaccines, in for instance *E. coli* and yeast, have not met desired protection levels, possibly due to their non-native glycan composition (Geldhof et al., 2007; Meyvis et al., 2008). In addition, several helminth glycoproteins that are investigated for their therapeutic potential rely on their glycan composition for their activity (Ryan et al., 2020; Bunte et al., 2022).

This review focuses on recent developments in glyco-engineering and possibilities to further improve glyco-engineering strategies in plants with special emphasis on glycoproteins from helminths. First, the wide diversity of helminth *N*- and *O*-glycans will be described. Second, reconstitution of various helminth glycoforms by using different glyco-engineering strategies in plants will be described. Finally, a perspective will be given on applications and possible future directions to produce non-human glycoproteins with tailor-made glycans.

## Diversity of Helminth Glycans

**FIGURE 2:**
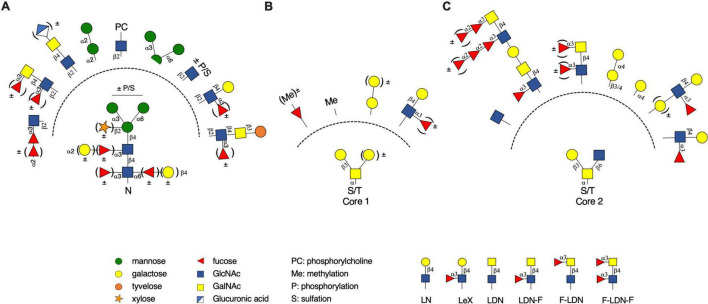
Diversity of helminth glycans. **(A)** A selection of *N*-glycan motifs found on different helminth glycoproteins. Besides immature mannosidic glycans, helminth *N*-glycans can be substituted with different core residues or are extended with different glycan motifs in their antenna. Some modifications are shared with animals, whereas other modifications are quite unusual. **(B,C)** A selection of *O*-glycan motifs found on different helminth glycoproteins, which are divided between typical core 1 **(B)** and core 2 **(C)** *O*-glycans. The linkage between different monosaccharides has been indicated where possible.

During an infection, helminths secrete immunomodulatory products, such as small molecules, RNA species, glycolipids and glycoproteins, to evade the host immune system and facilitate chronic infection (Bunte et al., 2022). The interplay between helminth secretory products and the host is partially mediated *via* glycan-dependent mechanisms (Bunte et al., 2022). Helminth glycoproteins can be decorated by a myriad of different *N*- and *O*-glycans as illustrated in Figure 2. The composition of these glycans differs between helminth species that belong to the distinct phyla Platyhelminthes (cestodes and trematodes) or Nematoda (nematodes). Furthermore, the glycan composition can differ between developmental stages and sexes within a single species (Smit et al., 2015; Hokke and van Diepen, 2017).

### Mannosidic-Type *N*-Glycans

All helminth species possess glycoproteins with mannosidic glycans. However, the abundance and diversity of the mannosidic glycans is species-dependent (Hokke and van Diepen, 2017). Mannosidic *N*-glycans can range from high-mannose type (Man7-9) to Man5 and paucimannosidic *N*-glycans (Man3). In addition, Man5 or Man3 *N*-glycans can carry a single branched GlcNAc residue with or without further branch extensions (hybrid or single-branched *N*-glycans, respectively). Several helminth species, like the trematode *Fasciola hepatica* and nematode *Haemonchus contortus*, contain even shorter *N*-glycans, where additional mannoses are trimmed from the Man3 structure resulting in Man2 glycan structures (Paschinger and Wilson, 2015; Garcia-Campos et al., 2016; Ravidà et al., 2016).

### *N*-Glycan Core Modifications

Various *N*-glycan core modifications have been observed in helminth species and in different developmental stages of a single species (Hokke and van Diepen, 2017). The main modification of the core is fucosylation. In early developmental stages of the trematode *Schistosoma mansoni*, *N*-glycans have an α1,3-fucose on their core. In later stages a core α1,6-fucose was found in the presence or absence of core α1,3-fucose. More densely fucosylated *N*-glycan cores were observed in adults of the nematode *H. contortus*. A trifucosylated core, with an α1,3-fucose attached to the distal core *N*-acetyl-glucosamine (GlcNAc) and two core fucoses (α1,3 and α1,6) attached to the proximal core GlcNAc, is the dominant glycan species on the H11 glycoprotein of *H. contortus* (Haslam et al., 1996). Additionally, galactosylation of the distal core α1,3-fucose and proximal core α1,6-fucose has been observed in *H. contortus* (Haslam et al., 1996; Hokke and van Diepen, 2017). *N*-glycan core xylosylation is uncommon in helminths, but has been observed in *S. mansoni* larval stages during the first three to 6 days after infection of humans, and in mature eggs and miracidia (Smit et al., 2015).

### Branching and Elongation of *N*-Glycans

The degree of *N*-glycan branching is also highly variable between different helminth species and between different developmental stages (Smit et al., 2015; Hokke and van Diepen, 2017). Complex tri- or tetra-antennary *N*-glycans are often extended with β1,4-linked galactose or *N*-acetyl-galactosamine (GalNAc) as part of a LacNAc (LN) or LacdiNAc (LDN) motif (Figure 2), respectively (Haslam et al., 2000; Smit et al., 2015). In several helminth species these LN and LDN motifs are further substituted with α1,3-fucose linked to a GlcNAc (part of a Lewis X or LD*N*-F motif) and is frequently found in the antennae of *N*-glycans. Additional α1,2-fucoses can be attached to the α1,3-linked fucoses in the antennae to form difucosylated glycan structures, as found on the *N*-glycans of SmVAL-9 (Yoshino et al., 2014). Another terminal glycan structure, but less common, is a fucosylated terminal poly GlcNAc structure, which has been described for *Dirofilaria immitis* (Martini et al., 2019). In contrast to human *N*-glycans extension of antennary galactose with sialic acid is never observed in helminths.

### Unusual *N*-Glycan (Non-)carbohydrate Moieties

Within the helminth glycome there are several unusual modifications found on *N*-glycans. The non-carbohydrate moiety phosphorylcholine (PC) was attached to antennary GlcNAc residues or distal core GlcNAc of *N*-glycans in several helminth species (Pöltl et al., 2007; Paschinger et al., 2012). PC can also be attached to GalNAc combined in a LDN(-F) motif, although this is less common (Wilson and Paschinger, 2016; Martini et al., 2019). PC is found in parasitic nematodes and cestodes, as well as in free-living nematodes such as *Caenorhabditis elegans* (Lochnit et al., 2000; Hokke and van Diepen, 2017). LDN motifs can also be extended with the unusual sugar moiety glucuronic acid that has been found on *N*-glycans of *Dirofilaria immitis* (Martini et al., 2019). Glucuronic acid is even found on antennae that are already modified with PC (Martini et al., 2019). Another rare sugar is tyvelose, which is found on *N*-glycans in *Trichinella spiralis* as an extension of LD*N*-F (Reason et al., 1994). Besides these uncommon moieties as part of helminth *N*-glycans other post-glycosylation modifications such as methylation, sulfation or phosphorylation were found on different helminth glycans (Ravidà et al., 2016; Jiménez-Castells et al., 2017).

### *O*-Glycans

Helminth glycoproteins can also harbor *O*-glycans and most of them are initiated by the addition of GalNAc (*O*-GalNAc glycans). *O*-glycosylation in helminths shows resemblance with mammalian *O*-GalNAc glycosylation but lacks variants that are capped with sialic acid. The structures encountered are similar to mammalian core 1 and core 2 *O*-glycan structures (Figure 2; Casaravilla et al., 2003; Hokke and van Diepen, 2017). Both cores can be elongated with a wide variety of glycan motifs, but the observed extensions can differ between core 1 and core 2 *O*-glycans (Hokke and van Diepen, 2017). Core 1 *O*-glycans can be elongated with poly-galactose (mainly α1-4 linked), Lewis X or a single fucose moiety as found in *Echinococcus multilocularis*, *S. mansoni* and *Toxocara canis*, respectively. Core 2 *O*-glycans can be elongated with terminal poly-galactose, Lewis X, F-LDN or LD*N*-F. Poly-fucosylated versions of LDN with α1,2-linked fucose (di-fucosylated and/or tri-fucosylated motifs) are found in *S. mansoni* (Smit et al., 2015). The possible combinations of core structures, elongations and terminal structures result in an array of different *O*-glycans (Talabnin et al., 2013; Smit et al., 2015; Hewitson et al., 2016). For several helminth species unique *O*-GalNac cores have also been observed. For *S. mansoni*, a core 1 *O*-glycan with additional β1,6-linked galactose and other extensions have been observed in several life stages (Smit et al., 2015). *H. contortus* has a rare *O*-GalNAc glycan, which is substitute with α1,3-linked galactose (core 8) (van Stijn et al., 2010). Not all helminth *O*-glycans are GalNAc-based glycans. *O*-glycans have been detected in *S. mansoni* and *Heligmosomoides polygyrus* that were initiated with an uncharacterized hexose (Jang-Lee et al., 2007; Hewitson et al., 2016). These rare *O*-glycans were further extended with fucose or another hexose residue. Helminth *O*-glycans can also be modified with post-glycosylation modifications such as methylation. Methylated *O*-glycans have been found on common core 1 and core 2 *O*-glycans of helminths (Hokke and van Diepen, 2017). Although the knowledge on the helminth *O*-glycome is relatively limited compared to the *N*-glycome, it is receiving more attention, possibly leading to the discovery of structures and post-glycosylation modifications previously unknown to the helminth *O*-glycome.

## Glyco-Engineering Helminth Glycans in Plants

**FIGURE 3:**
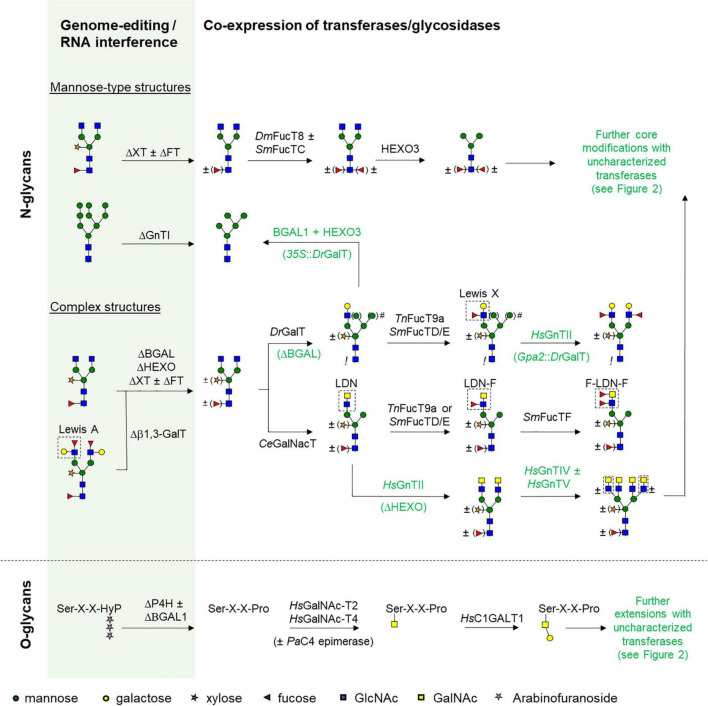
Strategies to engineer helminth glycans. Schematic presentation of glycan engineering strategies for the synthesis of helminth *N*- and *O*-linked glycans in plants. In the green column plant wild-type glycan structures are depicted. To optimize the plant glycosylation pathway for “helminthisation” several genes need to be silenced by RNA interference or knocked out by genome editing techniques and these are indicated with Δ. From these different starting points glyco-engineering strategies have been illustrated to synthesize different mannosidic *N*-glycan structures, more complex structures with different antennary glycan motifs, and initiation of core 1 *O*-glycans. After choosing an appropriate plant line as starting point (either wild-type, RNAi line or genome edited), glyco-engineering commences by c*o*-expression of different glycosyltransferases and/or glycosidases. The origin of each enzyme is given with two letter abbreviations for species names (Ce, *Caenorhabditis elegans*; Dm, *Drosophila melanogaster*; Dr, *Danio rerio*; Hs, *Homo sapiens*; Pa, *Pseudomonas aeruginosa;* Sm, *Schistosoma mansoni*; Tn, *Tetraodon nigriviridus*). No origin is given for plant endogenous enzymes. Enzymes: BGAL, β-galactosidase; HEXO, β-hexosaminidase; GnT, *N*-acetyl-glucosaminyltransferase; GalT, galactosyltransferase; GalNAcT, *N*-acetyl-galactosaminyl-transferase; FT, fucosyltransferase 11/12; FucT, fucosyltransferase; XT, xylosyltransferase. Other abbreviations or symbols: Asn, asparagine; Ser, serine; Thr, threonine; Pro, proline; Hyp, hydroxyproline; X, any amino acid; #, promoter strength for DrGalT determines the glycan composition, where control of expression with a dual 35S promoter results in hybrid glycans, but controlled expression with a weaker *Gpa2* resistance gene promoter results in single-branched glycans. !: core a1,3-fucose is absent upon over-expression of DrGalT.

The large variety in helminth *N*- and *O*-glycan composition largely affects glyco-engineering strategies in order to produce glycoproteins that could serve as immunomodulatory therapeutics or vaccine candidates. To study the effect of various glycoforms of these helminth proteins, a platform is needed that is capable of producing the desired glycoprotein with a defined glycan composition. A combination of strategies can be used to ultimately obtain a tailor-made glycoprotein that is identical to the native helminth glycoprotein, but can be different for each target species. In plants, this combination of strategies can include avoiding the addition of undesired plant glycan motifs (core α1,3-fucose, β1,2-xylose and/or Lewis A), addition of desired sugar residues by exogenous glycosyltransferases, and avoiding processing of glycans by endogenous glycosidases (Figure 3). Furthermore, it is important that newly introduced glycosyltransferases localize to the proper Golgi compartment. The localization of these enzymes to distinct Golgi compartments is determined by the cytoplasmic, transmembrane, and stem region (CTS region) of glycosyltransferases (Grabenhorst and Conradt, 1999; Sasai et al., 2001). If the localization is incorrect, glycosyltransferases are unable to function properly or the glycan is modified “too early,” which could interfere with subsequent modifications (Wilbers et al., 2017; Kallolimath et al., 2018). However, the latter option can also be exploited to alter the glycan in such a way that it is no longer recognized as substrate for glycosyltransferases later in the Golgi. This makes it possible to synthesize for instance mon*o*-antennary or hybrid *N*-glycans (Figure 3). Similarly, processing of glycans by endogenous glycosidases should in most cases be avoided, but sometimes the action of glycosidases could be beneficial to fine-tune a specific glycan composition (Figure 3).

### Mannosidic Type *N*-Glycans

To mimic the high-mannose type *N*-glycans (Man7-9) as found on many helminth glycoproteins, a strategy is needed that prevents maturation of these high-mannose *N*-glycans. The best known method to achieve this, is the addition of an ER retention signal, such as the C-terminal H/KDEL peptide sequence (Napier et al., 1992; Schouten et al., 1996). Retention of the glycoprotein in the late ER prevents the glycan from being modified by Golgi mannosidases. Alternatively, kifunensine (KIF), an α-mannosidase I inhibitor, has been used as a hydroponic treatment to increase the fraction of Man9 structures in *N. benthamiana* leaves (Krahn et al., 2017; Roychowdhury et al., 2018). Another inhibitor, swainsonine, has been used as additive in transgenic rice cultures to generate Man5 *N*-glycans by inhibition of α-mannosidase II (Choi et al., 2018). Man5 structures can also be created through the use of a *N*-acetyl-glucosaminyltransferase (GnT) I RNAi line of *N. benthamiana*, but this strategy also seemed to result in shorter Man3 and Man4 structures (Uthailak et al., 2021). Engineering toward specific mannosidic glycans remains challenging, as the necessary glyco-engineering strategies have not yet been developed or require additional optimization.

### *N*-Glycan Core Fucosylation and Xylosylation

Native plant *N*-glycans carry core a α1,3-fucose and β1,2-xylose, which are not always present on helminth *N*-glycans. Therefore, the inhibition of plant core α1,3-fucosyl- and β1,2-xylosyltransferases to prevent their addition to *N*-glycans is a prevalent achievement. Targeted interruption of core FucTs and XylTs has also been accomplished through RNA interference (RNAi) in a variety of plant species (Cox et al., 2006; Sourrouille et al., 2008; Strasser et al., 2008; Shin et al., 2011). For *N. benthamiana* this resulted in the ΔXT/FT RNAi line, which lacked plant-native *N*-glycan core modifications on the majority of the produced *N*-glycans (Strasser et al., 2008). The inability of RNAi to completely inactivate core FucT and/or XylT genes was later overcome through CRISPR/Cas9 technology in *Nicotiana benthamiana* (Jansing et al., 2019). Specific helminths, such as *S. mansoni*, do synthesize *N*-glycans that carry core α1,3-fucose with or without β1,2-xylose. The generation of individual knock-out plant lines for either core FucT or XylT genes by Jansing et al. (2019) may prove useful to obtain *N*-glycans that lack either α1,3-fucose or β1,2-xylose. Taken together, plant lines obtained through the different approaches may all be suitable for glyco-engineering of helminth *N*-glycans.

To synthesize core α1,6-fucosylated *N*-glycans in plants a core α1,6-fucosyltransferase (FucT) should be introduced, such as FucT8 from *Drosophila melanogaster*, *Homo sapiens* or *Mus musculus* (DmFucT8, HsFucT8, or MmFucT8, respectively) (Castilho et al., 2011a; Wilbers et al., 2016). In some cases, core α1,3-fucosylation is also desired. When using plants deficient in FucT and XylT activity, reintroduction of core α1,3-fucose is required, which can be achieved by c*o*-expression of an α1,3-FucT from *S. mansoni* (SmFucTC) or a hybrid *Zea mays* core α1,3-FucT with the CTS domain of *A. thaliana* FucT11 (Castilho et al., 2015; van Noort et al., 2020). An aspect that needs attention is glycosyltransferase inhibition that may occur upon simultaneous introduction of multiple FucTs. A strict order of fucosylation is observed for synthesis of double fucosylated *N*-glycan proximal cores of for instance *S. mansoni*, with α1,6-fucosylation preceding α1,3-fucosylation (Paschinger et al., 2005, 2019). Therefore, aberrant Golgi localization of transiently introduced (hybrid) FucTs should be carefully monitored to avoid disruption of the activity of introduced FucTs (van Noort et al., 2020).

### Introduction of Galactose-Extended *N*-Glycans

In plants, the only galactosylated native *N*-glycan structure is a Lewis A structure. Plants have shown to display the Lewis A glycan motifs on endogenous glycoproteins (Fitchette-Lainé et al., 1997; Strasser et al., 2007b) and in some cases on recombinant glycoproteins (Weise et al., 2007; Castilho et al., 2011b; Wilbers et al., 2016). The presence of Lewis A shows that plants have the machinery to add β1,3-galactose to glycans. Interestingly, β1,3-galactose is always seen in combination with α1,4-fucose in the Lewis A motif. The presence of plant Lewis A epitopes on recombinant glycoproteins can be completely abolished by the disruption of β1,3-galactosyltransferase 1 as has been demonstrated in moss (Parsons et al., 2012).

In contrast to any β1,3-galactose-extended glycans, helminth glycoproteins carry *N*-glycans that are extended with antennary β1,4-linked galactose (LN). The synthesis of L*N*-carrying *N*-glycans has been achieved in *N. tabacum* upon c*o*-expression of a hybrid β1,4-galactosyltransferase 1 (sialGalT) of *Danio rerio* or *H. sapiens* (Anusiem and Beetlestone, 1976; Hesselink et al., 2014). The original CTS domain of GalT was replaced with the CTS domain of *Rattus norvegicus* α2,6-sialyltransferase (sial). The sial-CTS domain translocates GalT to the *trans*-Golgi, as earlier addition of galactose can significantly hinder the activity of endogenous *N*-acetyl-glucosaminyltransferase II (GnTII), mannosidase II (α-Man II) and xylosyltransferase (XylT), leading to the synthesis of hybrid *N*-glycans (Johnson and Chrispeels, 1987; Bakker et al., 2001; Wilbers et al., 2017). In addition to localization, promotor strength can be exploited to avoid interference with the activity of certain glycosyltransferases to achieve the synthesis of a specific glycan (Wilbers et al., 2017; Kallolimath et al., 2018). For example, strong expression of sialGalT under control of a 35S promoter allows for the synthesis of hybrid *N*-glycans, whereas sialDrGalT expression under the control of the weaker Gpa2 promoter allows synthesis of single branched non-hybrid *N*-glycans (Figure 3).

Galactose-extended *N*-glycans are sometimes fucosylated (Lewis X), as is seen for *N*-glycans on omega-1 and IPSE/alpha-1 from *S. mansoni* (Wuhrer et al., 2006; Meevissen et al., 2010). Synthesis of Lewis X was achieved upon c*o*-expression of sialDrGalT and a hybrid α1,3-FucT9a (sialFucT9a) from *Tetraodon nigriviridus* (Rouwendal et al., 2009; Wilbers et al., 2017). More recently, the fucosyltransferases FucTD and FucTE from the parasite *S. mansoni* were identified as enzymes able to synthesize Lewis X, where SmFucTD and SmFucTE were most efficient (van Noort et al., 2020). The presence of predominantly monoantennary Lewis X-carrying *N*-glycans upon engineering suggests that introduction of a sialGalT still interferes with endogenous GnTII activity (Wilbers et al., 2017; van Noort et al., 2020).

While the synthesis of Lewis X is efficient, the synthesis of LN is not always efficient and depends on the protein under investigation (Strasser et al., 2009; Kriechbaum et al., 2020). For this reason, it was postulated that endogenous plant β-galactosidases (BGALs) along the secretory pathway could interfere with the synthesis of galactose-extended *N*-glycans. Recently, BGAL1 from *N. benthamiana* (homologous to BGAL8 from *A. thaliana*) has been characterized as an apoplast localized β-galactosidase and the major contributor to degalactosylation of both *N*- and *O*-linked glycans (including endogenous Lewis A). Impairment of NbBGAL1, through RNAi and CRISPR, resulted in a significant increase in galactosylation on various glycoproteins (Kriechbaum et al., 2020). Due to the redundancy of substrate specificity of the BGAL enzyme family and the number of β-galactosidases detected in apoplast fluid, it is likely that residual β-galactosidase activity may still lower the level of galactosylation (Dean et al., 2007; Buscaill et al., 2019). Therefore, additional research into remaining β-galactosidases and their ability to cleave *N*- and *O*-glycans is required to optimize glyco-engineering strategies for galactose containing *N*-glycans.

### Introduction of GalNAc-Extended *N*-Glycans

Helminth *N*-glycans often contain GalNAc as is seen in LDN and fucosylated LDN (Figure 2). Engineering of LDN carrying *N*-glycans requires the addition of a GalNAc on a terminal GlcNAc residue. Recently, LDN was synthesized on the *N*-glycans of *S. mansoni* kappa-5 in *N. benthamiana* by introduction of a *N*-acetyl-galactosaminyltransferase from *C. elegans* (CeGalNAcT) (Wilbers et al., 2017). GalNAc is never observed in plant glycans, and it was initially expected that incorporation of this sugar into *N*-glycans would require synthesis of UDP-GalNAc as substrate and facilitate its transport into the Golgi. However, it was observed that c*o*-expression of a C4 epimerase to enhance substrate availability as well as c*o*-expression of UDP-GalNAc transporters for improved Golgi localization was not required (Wilbers et al., 2017). This shows that, even though GalNAc is not native to plant *N*-glycans, UDP-GalNAc is present within the correct Golgi compartment.

The LDN motif can be further modified by expression of a α1,3-fucosyltransferase (α1,3-FucT; e.g., sialTnFucT9a, SmFucTD or SmFucTE) to yield a LD*N*-F motif (Wilbers et al., 2017; van Noort et al., 2020). Moreover, the glycan motif F-LD*N*-F could be synthesized upon c*o*-expression of these enzymes with SmFucTF. The possibility to synthesize fucosylated LDN with SmFucTD, SmFucTE and SmFucTF is a promising finding for the production of (fucosylated) LDN carrying helminth glycoproteins, such as kappa-5. From *S. mansoni.*

Similar to the synthesis of galactose-extended *N*-glycans, it seems that fucosylation of LDN, to obtain LD*N*-F, enhances the extension of *N*-glycans with GalNAc (Wilbers et al., 2017). Plant-native β-hexosaminidases (HEXOs) activity may limit the generation of LDN glycan motifs on kappa-5 in GalNAcT-expressing *Nicotiana benthamiana* (Alvisi et al., 2021). HEXOs are hydrolytic enzymes that are able to remove terminal GlcNAc and GalNAc residues from complex *N*-glycans, thereby generating typical plant paucimannosidic *N*-glycans (Shin et al., 2017; Alvisi et al., 2021). Plants harbor several HEXO homologs (HEXO1, HEXO2 and HEXO3) that differ by their substrate specificity and sub-cellular localization. HEXO1 and HEXO3 of *A. thaliana* and *N. benthamiana* are involved in the processing of plant *N*-glycans to paucimannosidic *N*-glycans, where HEXO1 is active in the vacuole and HEXO3 in the apoplast (Strasser et al., 2007a; Shin et al., 2017). *N*-glycans with non-endogenous GalNAc extended *N*-glycans can be processed by HEXO3 in the apoplast of *N. benthamiana*. In addition, HEXO2 was characterized as a membrane-bound enzyme specifically cleaving GalNAc residues from *N*-glycans (Alvisi et al., 2021). Unfortunately, targeted transient RNAi was not sufficient to improve LDN synthesis in plants (Alvisi et al., 2021). Future efforts should now focus on the generation of a NbHEXO knock-out plants to accelerate helminthisation of *N*-glycans in a plant expression system.

### Introduction of *N*-Glycan Branching

In contrast to plants, various helminth glycoproteins carry additional branches on their *N*-glycan structures. This was observed on tri- and tetra-antennary LDN(-F) carrying *N*-glycans of *S. mansoni* kappa-5, the tetra-antennary PC-glycans on ES-62 from *Acanthocheilonema viteae* and the tri- and tetra-antennary *N*-glycans with tyvelose of *T. spiralis* (Reason et al., 1994; Harnett et al., 2005; Meevissen et al., 2011). Plants can only synthesize up to two branches on their *N*-glycans, as plants lack *N*-acetyl-glucosaminyltransferases (GnT) IV and V (Sheshukova et al., 2016). Currently, the synthesis of *N*-glycans with LN, Lewis X, LDN and (F-)LDN(-F) is predominantly mon*o*-antennary (Wilbers et al., 2017; van Noort et al., 2020). C*o*-expression of exogenous GnTII from *A. thaliana* (AtGnTII) or *H. sapiens* (HsGnTII) could increase the occurrence of di-antennary *N*-glycans structures (Schneider et al., 2014; Dicker et al., 2016). In order to synthesize tri- or tetra-antennary *N*-glycans additional exogenous transferases are required, such as GnTIV and GnTV. Castilho and colleagues demonstrated that co-expression of GnTIV and GnTV from *H. sapiens* allows synthesis of tetra-antennary *N*-glycans (Castilho et al., 2011b). This strategy may also be used to produce helminth glycoproteins with tri- and tetra-antennary carrying *N*-glycans. A potential challenge for increasing the number of branches, is the possible activity of β-hexosaminidases on the initial GlcNAc, and possibly GalNAc extension, of each branch.

### Engineering Helminth *O*-Glycans

Helminth glycoproteins also have been shown to carry complex *O*-glycans (Hokke and van Diepen, 2017). The synthesis of *O*-glycans on helminth glycoproteins produced in plants is a next challenge that will allow the study of the role of these proteins in parasitism and their effectiveness as biopharmaceuticals. Just as with *N*-glycans, glyco-engineering of helminth *O*-glycans shows resemblance with humanization of the plant production platform. Most helminth *O*-glycans are initiated with *O*-GalNAc. *O*-GalNAc glycans are absent in plants, which allows for a bottom-up approach for the synthesis of such glycans. Two simultaneous, but independent studies have shown that *O*-GalNAc glycans can be synthesized, either by expression of GalNAcT2 and C1GalT1 leading to mucin core 1 *O*-GalNAc glycans (Castilho et al., 2012) or the combination of GalNAcT2, GalNAcT4and a GlcNAc C4-epimerase leading to *O*-GalNAc glycans (Yang et al., 2012). The synthesis of *O*-GalNAc glycans was possible despite hypothesized interference of prolyl-4-hydroxylases (P4H) and subsequent plant-native *O*-glycosylation in *N. benthamiana* (Monter*o*-Morales and Steinkellner, 2018). Plant native *O*-glycans could pose an issue in terms of immunogenicity when using plant-based pharmaceuticals (Yates et al., 1996; Leonard et al., 2005). In addition, plant native *O*-glycans could sterically hinder the synthesis of helminth *O*-glycans. A P4H1 knock-out in *Physcomitrella patens* prevents proline conversion into hydroxyproline (Hyp) on recombinant EPO (Parsons et al., 2013). In *N. benthamiana*, P4H1, P4H4, P4H9 and P4H10 have recently been identified as enzymes capable of forming hydroxyproline on recombinant proteins (Mócsai et al., 2021). Unfortunately, transient RNAi of these genes in *N. benthamiana* was insufficient to reduce P4H activity completely, due to redundancy within the substrate specificity of the P4H genes. However, it remains unknown to what extent plant-native glycosylation interferes with the substrate specificity of *O*-GalNAc glycans. It is anticipated that the effect of galactosidases and possibly hexosaminidases could be a bigger issue for the synthesis of mucin-type *O*-GalNAc glycans. BGAL1 from *N. benthamiana* has been shown to remove the terminal galactose from core 1 *O*-GalNAc glycans (Kriechbaum et al., 2020), whereas β-hexosaminidases can cleave off HexNAc residues in an elongated *O*-GalNAc glycan (Cheng et al., 2013). Therefore, more research is required into enzymes involved in *O*-glycan synthesis and hydrolysis, as this might be required for efficient production of native helminth *O*-glycosylated proteins.

## Perspective

Over the last two decades, “humanization” of the plant glycosylation pathway has shown that plants can efficiently produce various glycoproteins with engineered human *N*-glycans. But next to the focus on “humanization” of glycans, plants can be exploited to produce many other glycoproteins with tailor-made glycans. Enlarging the plant glyco-engineering toolbox for the synthesis of non-human glycans will fuel research to the function of specific glycan structures or glycoproteins with potential novel applications. In case of helminths, we described the possibilities to synthesize specific helminth *N*-glycans, which we refer to as “helminthisation” (Wilbers et al., 2017). Helminthisation of the plant glycosylation pathway will allow investigation into the function of specific glycan motifs and glycosyltransferases in parasite biology, development, and immunomodulation. Ultimately, the production of specific helminth glycan structures or helminth glycoproteins with a native glycan composition in plants can be used for development of vaccines, diagnostic tools, and novel biopharmaceuticals for the treatment of allergies and autoimmune diseases (Bunte et al., 2022).

Besides helminths, the search for useful glycoproteins can even be extended to other kingdoms. This includes potentially biologically active glycoproteins from marine animals or insects, which might have interesting applications as therapeutics (Caldwell and Pagett, 2010; Hykollari et al., 2018). Other possibilities include bi*o*-glycoprotein adhesives (Lutz et al., 2022), hydrogels (Böni et al., 2018; Zhong et al., 2018) and cryoprotectants (Tas et al., 2021; Dou et al., 2022). These various glycoproteins have specific glycosylation patterns that can be difficult to reconstitute in conventional expression systems and production of such glycoproteins could benefit from plant-based expression systems.

Plants offer a highly versatile and flexible expression system in terms of glyco-engineering, where glycans can be modified by introducing glycosyltransferases and/or glycosidases in a modular fashion (Figure 3). In addition, the choice of promoter strength can further increase the flexibility of the platform. For instance, we described that over-expression of β1,4-galactosyltransferases interferes with plant endogenous glycosyltransferases, but this offers opportunities to engineer hybrid or single-branched glycan structures as found in several helminth species. Although several helminth glycan motifs can be efficiently engineered in plants, other helminth glycan modifications cannot be synthesized yet in plants. In addition, little progress has been made on engineering the *O*-glycans observed in helminths, except for the initiation of core 1 *O*-glycans. For several helminth glycan modifications, the relevant glycosyltransferases, enzymes involved in substrate synthesis, and/or nucleotide transporters are currently still unknown. But, with the availability of many parasite genome sequences, plants offer an excellent platform for the functional characterization of novel parasite genes that are involved in the biosynthesis of helminth glycans, as was illustrated for fucosyltransferases of *S. mansoni* (van Noort et al., 2020).

Other techniques that greatly enhance the versatility of a plant-based glyco-engineering platform is the use of RNAi or genome editing techniques to counteract undesired glycan modifications by plant endogenous enzymes. With an expanding glyco-engineering toolbox, more and more enzymes are also being identified that should be targeted. The presence of recently identified glycosidases along the secretory pathway, such as BGAL1 and HEXOs (HEXO2 and HEXO3) in *N. benthamiana* (Kriechbaum et al., 2020; Alvisi et al., 2021), could pose a major hurdle for engineering helminth glycans that carry galactose- or GalNAc-extended glycans, respectively. With current developments in genome editing techniques in plants, the activity of these enzymes could easily be disrupted, but care should be taken with knocking out multiple of these genes at once. Some of these targets might be involved in vital biological processes in the plant, such as the synthesis or remodeling of endogenous *O*-glycans of cell wall glycoproteins (Petersen et al., 2021; Strasser et al., 2021), whereas others play a role in processing non-endogenous glycans and putatively play a role in plant defense responses of the plant (Buscaill et al., 2019; Alvisi et al., 2021).

Taken together, efforts to improve plants as production platform for glycoproteins with a defined glycan composition, offers possibilities to open-up new fields of research. Increased knowledge on engineering of the plant glycosylation pathways could assist other fields of research ranging from animal parasitology and immunology to plant sciences.

## Author Contributions

All authors listed have made a substantial, direct, and intellectual contribution to the work, and approved it for publication.

## Conflict of Interest

The authors declare that the research was conducted in the absence of any commercial or financial relationships that could be construed as a potential conflict of interest.

## Publisher’s Note

All claims expressed in this article are solely those of the authors and do not necessarily represent those of their affiliated organizations, or those of the publisher, the editors and the reviewers. Any product that may be evaluated in this article, or claim that may be made by its manufacturer, is not guaranteed or endorsed by the publisher.
